# Verbal Instructions and Motor Learning: How Analogy and Explicit Instructions Influence the Development of Mental Representations and Tennis Serve Performance

**DOI:** 10.3389/fpsyg.2020.00002

**Published:** 2020-02-07

**Authors:** Christopher Meier, Cornelia Frank, Bernd Gröben, Thomas Schack

**Affiliations:** ^1^Faculty of Psychology and Sports Science, Department of Sports Science, Sports and Education Research Group, Bielefeld University, Bielefeld, Germany; ^2^Faculty of Psychology and Sports Science, Department of Sports Science, Neurocognition & Action - Biomechanics Research Group, Bielefeld University, Bielefeld, Germany; ^3^Center of Excellence Cognitive Interaction Technology (CITEC), Bielefeld University, Bielefeld, Germany

**Keywords:** instruction, mental representation, long-term memory, chunking, junior tennis players, explicit learning, implicit learning

## Abstract

To better understand the benefits of using analogy and explicit instructions, the underlying cognitive mechanism remains to be explored. The concept of chunking provides a promising approach to the cognitive mechanism of instructions and can be approximated by analyzing athletes’ mental representations. The purpose of this study was to investigate the influence of analogy and explicit instructions on performance and the cognitive representations of the tennis serve in intermediate participants over the course of a 5-week training period. Junior tennis players (*N* = 44; *M* = 11.5 years) were tested on their tennis serve and, based on their initial performance and their individual error patterns, assigned to one of three groups: an analogy group (*N* = 15), an explicit group (*N* = 15), or a control group (*N* = 14). Their performance and their mental representation structures were assessed prior to and after the 5-week training period and again after a retention period of 14 days. Independent of group, findings demonstrated higher velocity from pretest to posttest. Participants in both the analogy and the explicit group showed enhanced accuracy over time and more functional mental representation structures. Thus, both analogy instruction and explicit instruction helped to structure mental representations in their long-term memory.

## Introduction

Verbal instructions have proven to be an effective way to help athletes learn movements and improve their performance (Hodges and Franks, 2002). Two types of verbal instruction have been researched extensively in the field of sports: Analogy instructions are pictorial descriptions based on movement experiences that are detached from their original context and transferred into new conditions. An analogy thus uses an image that already stands for a structurally similar movement and its effects, and evokes a mental image (Van Duijn et al., 2019b). As opposed to analogies, explicit instructions provide precise technical step-by-step instructions for movement execution including specific body positions or required movements (Schlapkohl and Raab, 2016; Meier et al., 2019).

In numerous studies, the effect of analogy instruction has been compared to the effect of explicit instruction on athletes’ performance. Both types of verbal instruction have been found to improve athletes’ performance (e.g., Masters et al., 2008; Lam et al., 2009a, b; Bobrownicki et al., 2015; Meier et al., 2019).

In adult novices, studies found performance improvements for both types of instruction, but results did not show performance differences between explicit instructions and analogies on single tasks in motor learning (e.g., Koedijker et al., 2007; Lam et al., 2009a, b; Van Duijn et al., 2019a). Results of secondary tasks yielded benefits for participants that were instructed by analogies (e.g., Liao and Masters, 2001; Koedijker et al., 2007, 2011; Lam et al., 2009b; Van Duijn et al., 2019a). However, the effects of analogies on performance under pressure seem “somewhat inconsistent” (Gröpel and Mesagno, 2019, p. 190). Regarding performance under pressure studies showed either no differences between analogy and explicit groups (e.g., Schücker et al., 2010, 2013; Bobrownicki et al., 2015) or more benefits when instructed by analogies (e.g., Liao and Masters, 2001; Law et al., 2003; Lam et al., 2009a). For adult intermediates, there seems to be no difference between analogy and explicit instructions in single tasks (Capio et al., 2019).

For younger athletes, results of a study demonstrated positive effects of analogies compared to explicit instructions investigating novice children on single tasks (Tse et al., 2017) as well as under pressure (Tse et al., 2017). In contrast, a study on intermediates showed better performances for explicit instructions (Schlapkohl et al., 2012). Schlapkohl et al. (2012) found that intermediates benefited more from explicit instructions than from a single analogy instruction, and explicit group’s performance did not decrease under a decision-making task (Schlapkohl et al., 2012).

Given the inconsistencies in findings to date, investigating athletes’ cognition might prove fruitful, as this might help to better explain the influence of different instructions on performance and learning. While studies to date have focused on performance improvements and motor learning, the impact of verbal instructions on athletes’ cognitive learning processes has not been subject to much empirical analysis. The concept of chunking (Poolton and Masters, 2014) may contribute to the explanation of the effectiveness and the ambiguous results in learning with different types of instruction. In the concept of chunking, “meaning associations emerge between recurring bits of information that are grouped together and coded as chunks. Over the course of practice, between-chunk associations develop and higher level chunks are compiled, until large amounts of the information pertaining to a task are hierarchically organized into a single representative chunk” (Poolton and Masters, 2014, p. 129). Particularly, analogy learning might be a promising way to chunk information more effectively. As the analogy provides already chunked information, “it represents a higher level of organization among the rules for the movement rather than explaining the task step-by-step” (Van Duijn et al., 2019a, p. 17). Additionally, analogies use an image that already stands for a structurally similar movement and its effects. Thus, information is processed with less cognitive effort.

So far, studies tested the concept of chunking in sequence learning tasks (e.g., Bellomo et al., 2018; for an overview, see Abrahamse et al., 2013) that, however, differ from learning complex motor skills with different types of instruction. Recently, Van Duijn et al. (2019a) investigated the effect of different types of instruction on cognitive processes by using electroencephalography (EEG). The authors examined differences in psychomotor efficiency between analogy, explicit instruction, and no instruction in novice adults practicing a hockey push-pass task. Van Duijn et al. (2019a) speculated that this higher efficiency of the analogy group might indicate chunking processes. However, this assumption cannot be concluded from their results directly.

One way to more directly address this chunking concept is to analyze mental representations of a complex action (e.g., Schack, 2004a, b, 2007, 2010; Schack et al., 2008). The cognitive architecture of complex actions refers to the interplay of higher levels of mental, and lower levels of sensorimotor, control, and representational systems, with mental representations playing a key role in motor control and learning (Schack, 2004a). The resulting biomechanical structure of movements is herein not considered independent of the sensory effects of the motor action, but rather is a result of the interplay across levels of action organization and, thus, is linked directly to cognitive–perceptual representations of the action. Accordingly, node points have been suggested to bundle movement sequences into representations of suitable steps for executing actions. More specifically, mental representations are believed to be structured with basic action concepts (BACs) as cognitive chunks for the control of actions (Schack, 2004b; Schack and Mechsner, 2006). It has been assumed that BACs are the mental counterparts to functional elementary components of complex movements and can be seen “as the cognitive chunking of postures and movement events related to common functions in the realization of action goals” (Schack et al., 2014, p. 2). BACs do not relate to behavior-related invariant properties of objects, but rather to perception-linked invariant properties of movements. “Mental representations are thought to comprise such representation units and their structural composition in relation to one another” (ibid.). The relationship between and the groupings of the BACs of a motor action determine the structure of a specific mental representation.

In numerous studies, mental representations of complex sport movements have been found to be dependent on expertise (e.g., Schack and Mechsner, 2006; Schütz et al., 2009; Land et al., 2013). In experts, the structure of such representations is more functional and more related to biomechanical principles than in novices. Schack and Mechsner (2006) investigated expert tennis players’ mental representations of the serve. The results demonstrated that the mental representation structures were well matched with the functional phases of the serve, whereas novices showed less functional structures (Schack and Mechsner, 2006).

Motor learning can be described as a change, modification, and development of action-related representation structures in long-term memory, resulting in more functional representations. Frank et al. (2013) investigated changes in both the motor performance and the mental representation structure of the golf putt in novices over the course of practice. They found that practice led to an improvement in performance that was accompanied by functional changes in mental representation structures. Similarly, mental representations have been found to change functionally with mental practice such as imagery (Frank et al., 2014, 2016, 2018b; Simonsmeier et al., 2018) and observation (Frank et al., 2018a) as well as in the context of expertise (e.g., Schack and Mechsner, 2006) and rehabilitation (Braun et al., 2007).

Although this body of evidence shows that learning is accompanied by functional changes in mental representation structures resulting from chunking processes, it remains unknown how different types of verbal instruction (i.e., analogy and explicit instruction) affect the mental representation structure of a complex action. If analogies and explicit instructions lead to improved performance through changes in underlying mental representations, then changes in representation structures should be evident after receiving such verbal instructions and practicing with them in mind.

Based on the issues identified in the previous sections, the current field study aims to investigate the influence of analogy and explicit instructions on outcome performance and cognitive structures (i.e., mental representations) of junior tennis players’ serve given an equal number of instructions according to individual error patterns (described in more detail below).

In previous research, participants in the analogy conditions have frequently been provided with one solitary analogy instruction, while participants in the explicit conditions have been provided with many explicit instructions (e.g., Lam et al., 2009a; Schlapkohl et al., 2012; Schücker et al., 2013). From this, it is difficult to determine whether differences in performance were due to different amounts of instructions used (probably leading to differences in attentional load) or due to differences that could be attributed to the instructions themselves (Bobrownicki et al., 2015, 2018). In this study, therefore, an equal number of explicit and analogy instructions was used.

The aim of this study was 2-fold: First, we compared the velocity and accuracy of the participants’ serve influenced by the two types of verbal instruction. We expected performance of the tennis serve to differ between the two groups depending on the instructions given. Based on the partially inconsistent results of previous studies of intermediate athletes, we did not predict whether the intermediate tennis players’ serve performance would benefit more when they receive either explicit or analogy instructions. Second, we investigated whether and how mental representation structures would be influenced by the two kinds of verbal instruction. We predicted that an improvement in performance would be accompanied by a (functional) structuring of mental representations.

## Materials and Methods

### Participants

In this field study^1^, 44 junior tennis players (*n* = 44; *M* = 11.5 years, *SD* = 1.65; 15 females, 29 males) who were in a training base of their region or played at such a competitive level were involved. Participants practiced on a regular basis two to three times a week. The players were assigned to one of three groups: analogy instruction (*n* = 15; *M* = 11.13 years, *SD* = 1.24; 8 females, 7 males), explicit instruction (*n* = 15; *M* = 11.20 years, *SD* = 1.74; 4 females, 11 males), or control (*n* = 14; *M* = 12.21 years, *SD* = 1.80; 3 females, 11 males). Participants were allocated to a group according to their serve performance (accuracy and velocity)^2^ on a pretest as well as on their serve’s individual error patterns (for details, see instructions). Distribution of participants to the groups generally was equal in terms of performance, age, and sex. All the parents gave their informed consent before the participants started their involvement in the study, which was in accordance with local ethical guidelines.

### Apparatus and Task

#### Performance

The participants performed serves from the deuce side of an outdoor tennis court to a target point (0.7 meters from the serve line and 0.7 meters from the center serve line). Left-handers served from the other side. Accuracy and velocity are essential to serve performance in tennis. A coordinate system of measuring tapes was placed to determine the exact point of impact. Accuracy was recorded by measuring the exact point of impact (Hernández-Davo et al., 2014) of the ball with its *x* and *y* coordinates to identify the mean radial error of all trials. Each coordinate was recorded, after determining the point of impact visually.

To determine the velocity of the serves, we used a professional radar gun (Stalker Radar S Pro II). Radar placement to measure the velocity of the ball is important for obtaining accurate results (Tubez et al., 2017). First, the angle between the radar sight and the ball trajectory should be as small as possible. Second, the radar should be placed behind the starting point to measure the maximal velocity of the ball upon exiting the racket (Tubez et al., 2017). Thus, the radar gun was located at a height of 2.5 and 3 m behind the player and was oriented toward the target.

#### Structural Dimensional Analysis of Mental Representation (SDA-M)

To analyze mental representation structures of the tennis serve, an SDA-M was conducted. More specifically, the SDA-M proceeds in four steps: (1) a split procedure delivering a distance scaling between the BACs of a suitably predetermined set, (2) a hierarchical cluster analysis used to outline the structure of the given set of BACs, (3) a factor analysis revealing the dimensions in this structured set of BACs, and (4) an analysis of invariance within and between groups in order to compare different cluster solutions (Schack, 2012).

More specifically, in order to determine distances between BACs in memory, mental representation structure was assessed by way of a split procedure, the first step of the SDA-M described below. First, the experimenter introduced the split procedure and explained each of the 11 BACs in a randomized order without demonstrating execution of the movements. Next, the experimenter showed on a computer screen the general instructions for completing the split procedure. The participants were asked to decide whether or not the BACs presented on the screen belonged together during movement execution.

The split procedure was performed in front of a computer displaying the BACs of the tennis serve. The BACs were explained verbally. During the split procedure, one of the BACs was located in the anchor position and was permanently displayed while all other BACs were displayed in a randomized order. For each pair of BACs displayed (i.e., anchor BAC plus additional BAC), the participants indicated whether or not the two BACs shown belonged together during the movement execution (yes/no decisions). Once all BACs had been assessed in relation to the anchor BAC, that is, after a decision had been made for each pair of BACs, a different BAC took over the anchor position and the procedure was repeated. The split procedure ended once each BAC had been in the anchor position (Schack, 2012).

In this study, we employed BACs outlined by Schack and Mechsner (2006). From a functional and biomechanical perspective, a tennis serve consists of three distinct phases (Göhner, 1992, 1999): pre-activation, strike, and swing. In the pre-activation phase, body and ball are brought into position, and tension energy is exerted to prepare the strike. Schack and Mechsner (2006) identified the following BACs in this phase: (1) ball throw, (2) forward movement of pelvis, (3) bending the knees, and (4) bending the elbow. In the strike phase, energy is transferred to the ball. The following BACs are involved in this phase: (5) frontal upper body rotation, (6) racket acceleration, (7) whole body stretch motion, and (8) hitting point. In the final, swing phase, the body is prevented from falling and the racket movement decelerates after the strike. The following BACs are involved in this phase: (9) wrist flap, (10) forward bending of the body, and (11) racket follow-through.

### Design and Procedure

Accuracy, velocity, and mental representation structures of the tennis serve were measured before and after the training period. In addition, the two instruction groups were tested after a retention interval of 2 weeks to test whether improvements would persist over time, and thus reflect learning.

On each day of testing, participants’ tennis serve performance and mental representation structure were measured in an indoor tennis court. First, to determine changes in the structure of mental representations, the participants completed the split procedure described above. Second, each player had a 10-min warm-up activity and then performed eight warm-up serves. The participants then performed two rounds of 15 serves, aiming at a target located in the service box. The players were instructed to serve the ball as accurately and as fast as possible (e.g., Hernández-Davo et al., 2014).

The participants practiced their tennis serve during a training period of 5 weeks. During that time, the participants performed serves with individualized instructions. Instructions were given four times (every 20 serves). Additionally, participants were reminded after 10 serves to follow the given instructions. Each week, the tennis players performed 80 tennis serves twice a week, resulting in a total of 160 tennis serves per week, and 800 serves overall during acquisition phase. The control group performed a similar number of serves without receiving any instruction.

#### Instructions

The ability to use instructions effectively during practice depends heavily on whether the instructions fit with the task as well as with the athlete, especially with regard to analogies. We account for this in two ways: First, and prior to the study, experts (five tennis coaches with B-/A-level and considerable experience in tennis training) were asked for analogies they used in their own training sessions. Then, to develop content-valid instructions for the tennis serve, a discussion was conducted with coaches and researchers to determine adequate analogies for the different movement phases of the tennis serve.

Second, assuming that instructions vary with regard to different serve’s error patterns, we used an individualized approach. Based on pretest data and assessments of athletes’ coaches and researchers, we identified the main error patterns in each participant’s tennis serve. Thus, each player received individualized instruction corresponding to the specific movement-related problem(s) he or she exhibited on the pretest. More precisely, if a problem during the loading phase was identified, a participant received an instruction related to this specific problem area and not to the overall movement.

Table 1 presents the different instructions. In a functional perspective, the deceleration phase has no influence on performance outcome as the ball has already left the racket. Accordingly, no instructions regarding deceleration phase are presented in Table 1.

**TABLE 1:** Analogy Instructions and explicit instructions for each movement phase [adapted from Meier et al. (2019)].

Phase	BAC	Analogy instruction	Explicit instruction
Pre-activation	(1) Ball throw	–“Imagine your racket is the hand of a watch that goes	–“Move the racket first down and then up.”
	(2) Forward movement of pelvis	counterclockwise from 9:00 to 3:00.”	–“Raise the ball with your arm outstretched.”
	(3) Bending the knees	–“Imagine your ball going up in a narrow elevator and	–“Move the pelvis forward.”
	(4) Bending the elbow	getting off at front height on the top floor.”	–“Tilt your upper body increasingly backward.”
		–“Imagine getting into the position of an archer just	
		before an arrow is fired.”	
		–“Imagine getting in a trophy-position.”	
Strike	(5) Frontal upper body rotation	–“Imagine you are tensing as a spring which then	–“Push yourself off the ball and stretch your knees first,
	(6) Racket acceleration	releases.”	then hip, stomach, chest, shoulder and arm muscles
	(7) Whole body stretch motion	–“Imagine the racket being thrown into the ball.”	one after the other.”
	(8) Hitting point	–“Imagine you want to look at your watch at the hitting	–“Accelerate from bottom to top.”
		point.”	–“Turn your racket through your wrist just before the
		–“Imagine your ball is an elevator in a high-rise building	hitting point.”
		and your racket wants to get in on floor 15.”	–“Hit the ball at the highest possible point.”

#### Manipulation Check

To determine whether the players performed as instructed and to ensure that the correct problem area was addressed, the participants in the two groups that received verbal instructions completed a questionnaire after the first, third, and fourth practice sessions. On the questionnaire, they indicated on six-point Likert scales (1 = very difficult, 6 = very easy) how easy it was to follow the instructions and how easy it was to imagine the movements involved in the tennis serve. In addition, they were asked to mention any problems they experienced during training (Frank et al., 2016).

### Data Analysis and Dependent Variables

#### Performance

Mean radial error (MRE) was calculated to determine the distance between the target and where, on average, the balls landed (Hancock et al., 1995). The location of a shot in relation to the target provides information on the height and direction of bias. To investigate the representation of a typical position of a block of attempts, we used the centroid. The centroid is a point whose coordinates are given by the average *x* value and average *y* value of the shots being included. From this point, a participant’s centroid radial error (SRE) represents the radial distance of the centroid from the target and is a measure of magnitude of bias over a set of attempts from a single participant (Hancock et al., 1995)^3^.

To determine changes in performance from the pretest to the posttest, a 3 (analogy, explicit, control) × 2 (pre, post) repeated-measures analysis of variance (ANOVA) was calculated (IBM SPSS Statistics 25) for each of the dependent variables. Motor learning was examined using a 2 (analogy, explicit) × 3 (pre, post, retention) repeated-measures ANOVA. *Post hoc* analyses (independent group and pairwise comparisons) were conducted employing a Bonferroni correction.

#### Mental Representation Structures

Mental representation structures were analyzed by calculating mean group dendrograms via cluster analyses (Schack, 2012). For all analyses, an alpha-level of α = 0.05 was chosen with a critical value of *d*_*crit*_ = 3.41. BACs linked below the critical value resulted in clusters, thus groupings of BACs. The lower the value of a link between two items, the shorter the distance was between the related BACs in long-term memory. To compare differences between clusters, analyses of invariance were conducted. According to Lander (1991), two clusters are variant, which is significantly different for λ < 0.68, while two clusters are invariant for λ ≥ 0.68.

Furthermore, the adjusted rand index (ARI) ranks the similarity of the groups’ mental representations to that of an expert player (Santos and Embrechts, 2009). The ARI ranges as an index of similarity from −1 to 1. A value of −1 indicates that clusters differed. A value of 1 indicates that two clusters were the same. Between these two extreme indices, the value ranks similarity between two clusters.

The structure of an expert’s mental representation of the phases of the tennis serve (cluster 1: BACs 1, 2, 3, 4; cluster 2: BACs 5, 6, 7, 8; cluster 3: BACs 9, 10, 11) was chosen as a reference structure for the analyses of the participants’ mental representations. This reference structure corresponds to the expert structure presented in Schack and Mechsner (2006).

## Results

### Manipulation Check

Results of the manipulation check^4^ were analyzed to determine whether participants in the analogy group and the explicit group adhered to the instructions they received during the training period. None of the participants reported having problems understanding the verbal instructions during the training sessions. The mean score (on a scale from 1 = very difficult to 6 = very easy) of the participants in the analogy group was 4.70 (*SD* = 1.02) and that of those in the explicit group was 4.92 (*SD* = 1.21), indicating that participants were able to imagine the movement of the tennis serve from the instructions they were given. The mean score of the understandability of the instructions for the participants in the analogy group was 5.30 (*SD* = 0.67) and that for the participants in the explicit group was 5.12 (*SD* = 0.79), indicating that the participants found the instructions easy to follow. Hence, the participants were able to understand the instructions and to transform them into movements during practice of the tennis serve, which was essential for the validity of the data analyses.

### Performance

#### Accuracy

**FIGURE 1:**
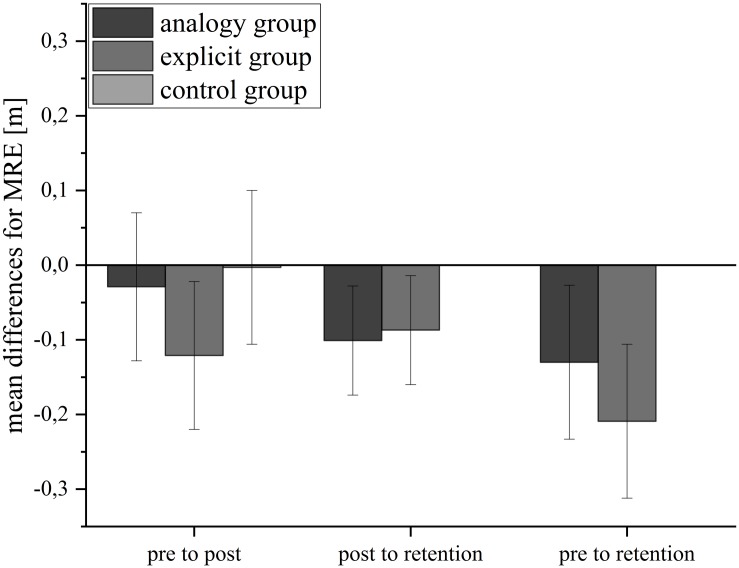
Mean differences for mean radial error (MRE) in meters for the three groups from pretest to posttest, posttest to retention test, and from pretest to retention test. Error bars represent standard errors.

Figure 1 illustrates the MRE of the serve performance for all groups. In Table 2, the descriptive statistics are shown. To measure development in accuracy from the pretest to the posttest, a 3 (analogy, explicit, control) × 2 (pre, post) ANOVA was conducted for MRE as a dependent variable. An ANOVA yielded no significant main effect of time and no interaction effect between time and group on the MRE.

**TABLE 2:** Descriptive statistics for MRE in meters across the pretest, posttest, and retention test for the three groups.

Group	Pretest		Posttest		Retention test	
	*M* (*SD*)	95% CI	*M* (*SD*)	95% CI	*M* (*SD*)	95% CI
**MRE**						
Analogy	1.87	[1.63,	1.84	[1.63,	1.74	[1.56,
(*n* = 15)	(0.43)	2.10]	(0.34)	2.05]	(0.28)	1.91]
Explicit	1.76	[1.53,	1.64	[1.43,	1.55	[1.38,
(*n* = 15)	(0.46)	2.00]	(0.43)	1.85]	(0.38)	1.73]
Control	1.81	[1.56,	1.80	[1.59,	–	–
(*n* = 14)	(0.48)	2.06]	(0.43)	2.03]		

*M, mean; SD, standard deviation; CI, confidence intervals.*

The MRE from the pretest to the posttest and retention test was examined by conducting a 2 (analogy, explicit) × 3 (pre, post, retention) within-subject ANOVA. The results indicated a significant main effect of time on the MRE, *F*(2,56) = 3.39, *p* = 0.041, $\eta_{\text{p}}^{2}$ = 0.108, but no significant interaction effect between time and group.

**FIGURE 2:**
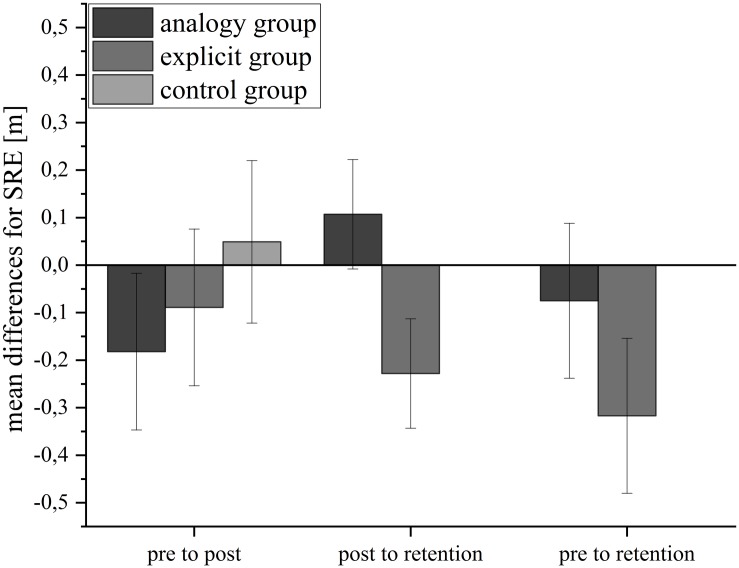
Mean differences for subject-centroid radial error (SRE) in meters for the three groups from pretest to posttest, posttest to retention test, and from pretest to retention test. Error bars represent standard errors.

Figure 2 illustrates the SRE of the serve performance for all groups. In Table 3, the descriptive statistics are shown. The analysis of SRE revealed no significant effects.

**TABLE 3:** Descriptive statistics for SRE in meters across the pretest, posttest, and retention test for the three groups.

Group	Pretest		Posttest		Retention test	
	*M* (*SD*)	95% CI	*M* (*SD*)	95% CI	*M* (*SD*)	95% CI
**SRE**						
Analogy	1.08	[0.81,	0.90	[0.66,	1.01	[0.79,
(*n* = 15)	(0.53)	1.36]	(0.46)	1.15]	(0.34)	1.23]
Explicit	0.96	[0.69,	0.87	[0.63,	0.65	[0.43,
(*n* = 15)	(0.51)	1.24]	(0.48)	1.12]	(0.47)	0.86]
Control	0.96	[0.67,	1.01	[0.76,	–	–
(*n* = 14)	(0.56)	1.25]	(0.48)	1.26]		

*M, mean; SD, standard deviation; CI, confidence intervals.*

#### Velocity

**FIGURE 3:**
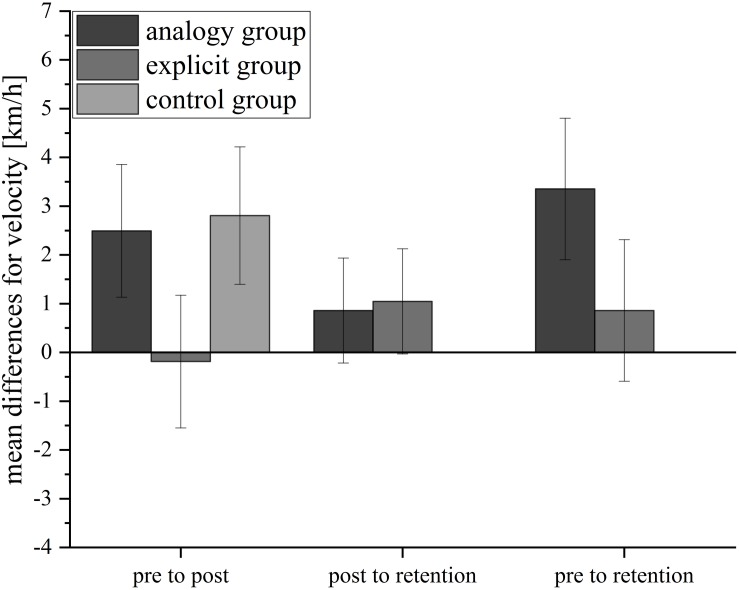
Mean differences for velocity in km/h for the three groups from pretest to posttest, posttest to retention test, and from pretest to retention test. Error bars represent standard errors.

Figure 3 illustrates the velocity of the serve performance for all groups. In Table 4, the descriptive statistics are shown. To measure development in velocity from the pretest to the posttest, a 3 (analogy, explicit, control) × 2 (pre, post) ANOVA was conducted for velocity as a dependent variable. The results indicated a significant main effect of time, *F*(1,41) = 4.60, *p* = 0.038, $\eta_{\text{p}}^{2}$ = 0.101, but no significant interaction effect between time and group.

**TABLE 4:** Descriptive statistics for velocity in meters across the pretest, posttest, and retention test for the three groups.

Group	Pretest		Posttest		Retention test	
	*M* (*SD*)	95% CI	*M* (*SD*)	95% CI	*M* (*SD*)	95% CI
**Velocity**						
Analogy	105.4	[95.2,	107.9	[97.6,	108.8	[98.3,
(*n* = 15)	(16.8)	115.7]	(19.4)	118.3]	(19.4)	119.3]
Explicit	111.7	[101.5,	111.5	[101.2,	112.6	[102.1,
(*n* = 15)	(20.3)	122.0]	(19.8)	121.9]	(20.2)	123.1]
Control	111.7	[101.1,	114.5	[103.8,	–	–
(*n* = 14)	(21.7)	122.3]	(20.5)	125.3]		

*M, mean; SD, standard deviation; CI, confidence intervals.*

The velocity from the pretest to the posttest and retention test was examined by conducting a 2 (analogy, explicit) × 3 (pre, post, retention) within-subject ANOVA. The ANOVA yielded no significant effects for velocity.

### Mental Representation Structures

Figures 4–11 represent groups’ cluster solutions. At pretest, groupings were mostly non-functional (i.e., clusters are comprised of BACs of different phases) and thus differed from a functional reference structure (cluster 1: BACs 1, 2, 3, 4; cluster 2: BACs 5, 6, 7, 8; cluster 3: BACs 9, 10, 11).

**FIGURE 4:**
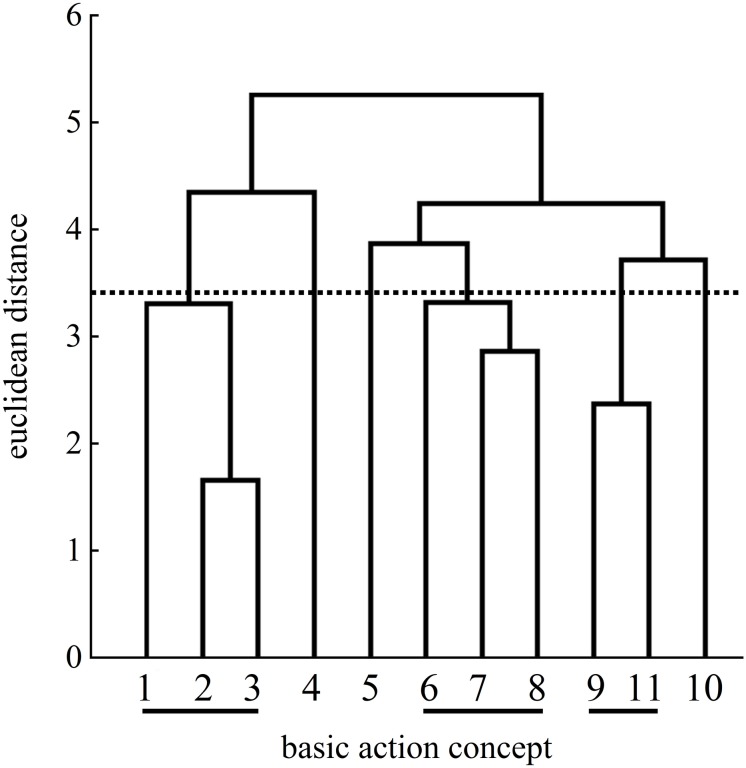
Mean group dendrogram of the analogy group’s (*n* = 15) tennis serves on the pretest. The numbers on the *x*-axis represent the BACs; the numbers on the *y*-axis represent Euclidean distances. The lower the link between the BACs was, the shorter the Euclidean distance. The horizontal dotted line marks *d*_*crit*_ for a given α-level (*d*_*crit*_ = 3.41; α = 0.05): Links between the BACs above the line are considered unrelated; BACs (1) ball throw, (2) forward movement of pelvis, (3) bending the knees, (4) bending the elbow, (5) frontal upper body rotation, (6) racket acceleration, (7) whole body stretch motion, (8) hitting point, (9) wrist flap, (10) forward bending of the body, and (11) racket follow-through.

Examination of the analogy group’s posttest mean dendrogram indicated significant differences between the pretest and the posttest (λ = 0.44) as well as between the pretest and the retention test (λ = 0.43). The cluster solutions of the posttest and the retention test were considered the same statistically (λ = 0.70). The mean dendrogram of the analogy group was less similar to that of an expert at posttest (ARI = 0.41) compared to the pretest (ARI = 0.56), but became more similar to that of the reference mental representation structure at retention test (ARI = 0.60).

**FIGURE 5:**
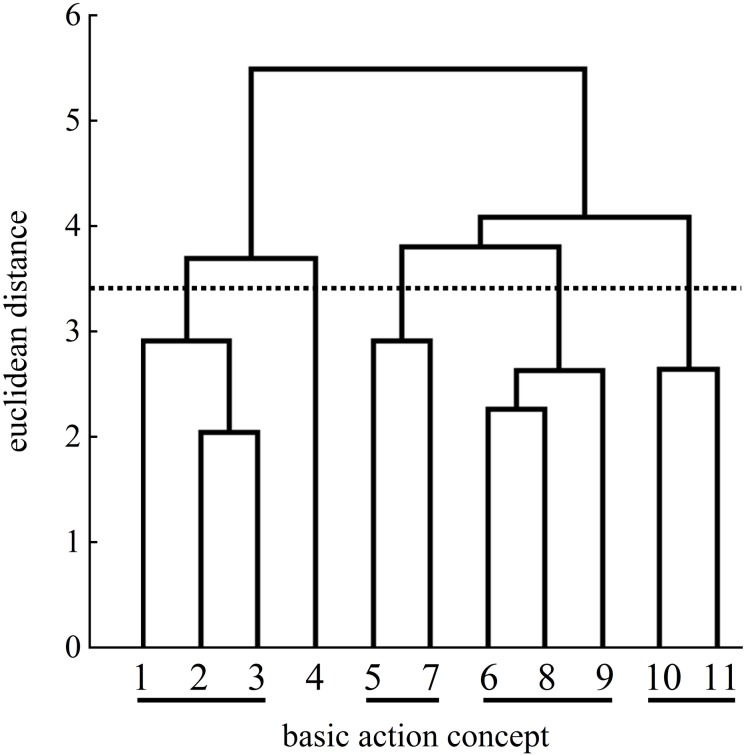
Mean group dendrogram of the analogy group’s (*n* = 15) tennis serves on the posttest.

**FIGURE 6:**
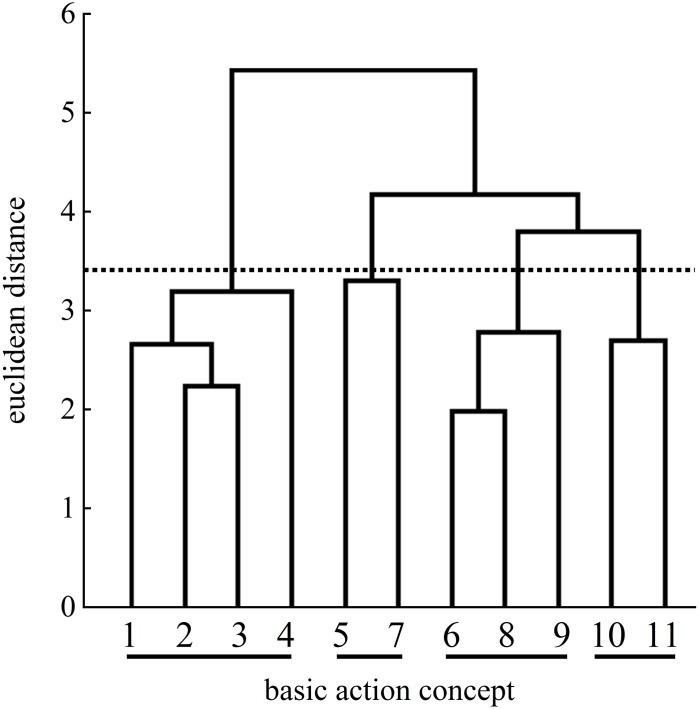
Mean group dendrogram of the analogy group’s (*n* = 15) tennis serves on the retention test.

More precisely, Figure 5 shows an increased number of clusters at the posttest. One cluster of BACs relates to the preparation of the tennis serve: BAC 1 (ball throw), BAC 2 (forward movement of pelvis), and BAC 3 (bending the knees). The same cluster was found at the pretest (Figure 4). A second cluster of the posttest was related to the striking phase: BAC 5 (frontal upper body rotation) and BAC 7 (whole body stretch motion). A third cluster was related to the striking phase: BAC 6 (racket acceleration), BAC 8 (hitting point), and BAC 9 (wrist flap) of the final, swing phase. Lastly, a fourth cluster was related to the final, swing phase: BAC 10 (forward bending of the body) and BAC 11 (racket follow-through). Although the dendrograms of the analogy group for both the posttest and retention test (Figure 6) showed a four-cluster solution, one slight difference was found between the posttest and retention test dendrograms. For the first cluster, namely, for the preparation phase, the cluster of the posttest consisted of BACs 1, 2, and 3, but at the retention test, BAC 4 was also linked with BACs 1, 2, and 3, representing all BACs of the preparation phase.

For the explicit group, statistical analysis of invariance showed a significant difference between the pretest and posttest (λ = 0.53) as well as between the pretest and the retention test (λ = 0.51). The clusters of the posttest and the retention test were statistically different (λ = 0.51). When compared to the mental representation structure of an expert, the mean dendrograms of the explicit group’s mental representation structures developed from the pretest (ARI = −0.04) to the posttest (ARI = 0.07) and to the retention test (ARI = 0.34).

**FIGURE 7:**
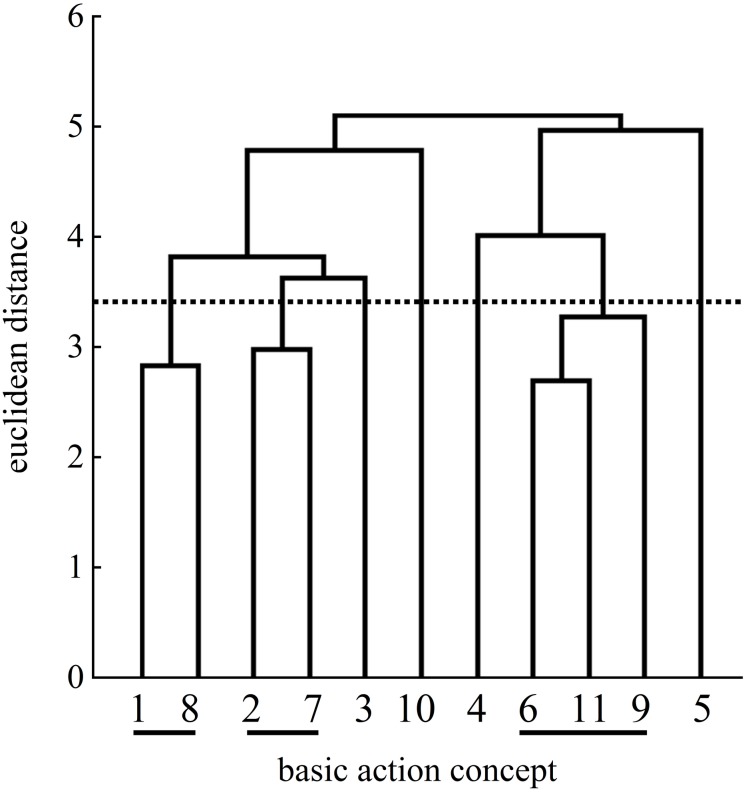
Mean group dendrogram of the explicit group’s (*n* = 15) tennis serves on the pretest.

**FIGURE 8:**
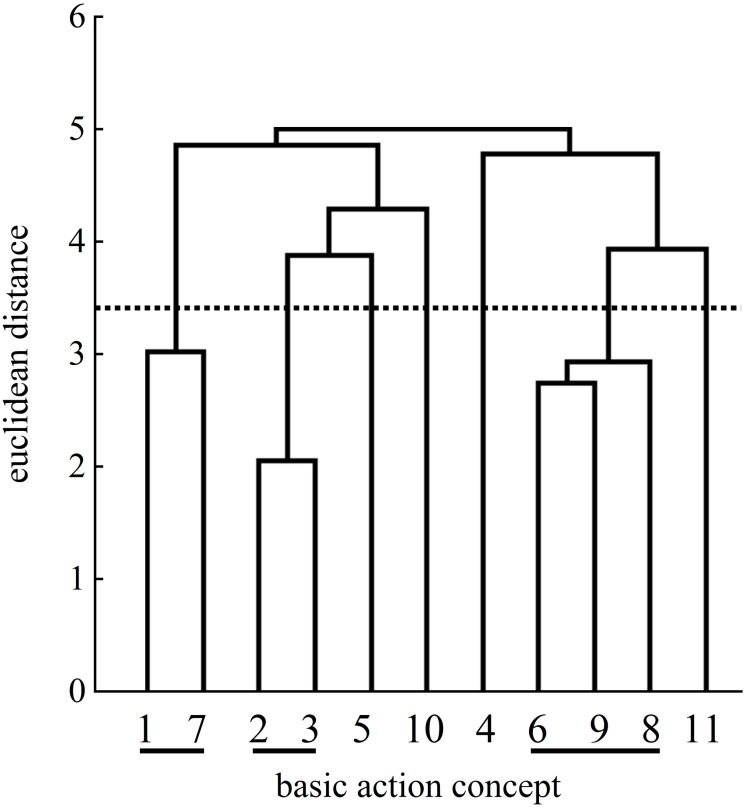
Mean group dendrogram of the explicit group’s (*n* = 15) tennis serves on the posttest.

**FIGURE 9:**
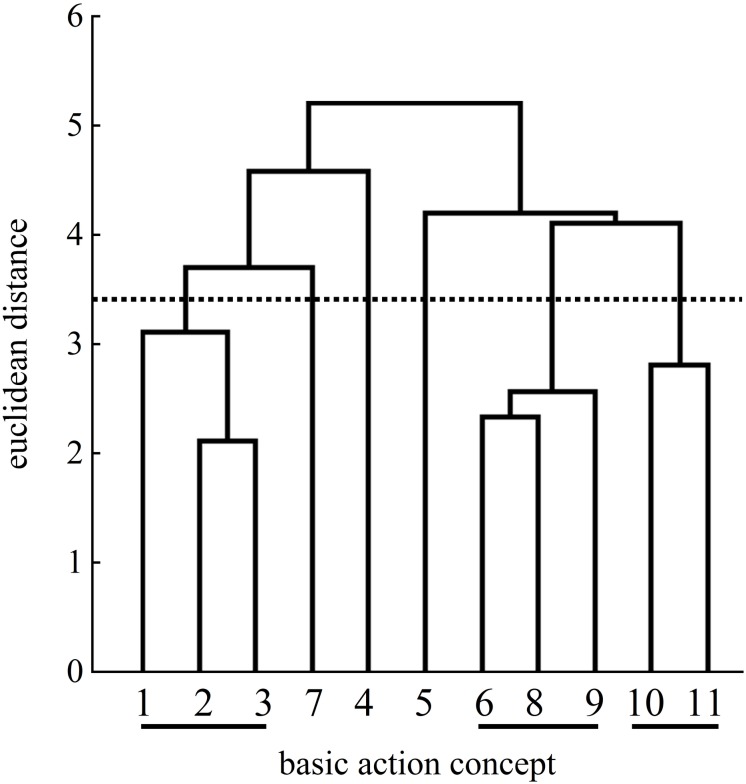
Mean group dendrogram of the explicit group’s (*n* = 15) tennis serves on the retention test.

In detail, the explicit group’s posttest mean dendrogram revealed the same number of clusters (Figure 8) as on the pretest (Figure 7). At the pretest, the three clusters of BACs indicated no functional units of the mental representation structure. Although the posttest dendrogram displayed an identical number of clusters, the connected BACs differed. One cluster included BAC 1 (ball throw) and BAC 7 (whole body stretch motion). The second more functional cluster was related to the preparation phase with BAC 2 (forward movement of pelvis) and BAC 3 (bending the knees). Lastly, the third cluster was related to BACs 6 (accelerating the racket), 9 (wrist flap), and 8 (hitting point). Although the mean dendrograms for the posttest and the retention test (Figure 9) both displayed three clusters, the explicit group’s mean dendrograms revealed an increase in the number of functional connections between the posttest dendrogram and the retention test dendrogram. For the first cluster of BACs, namely, those related to the preparation phase, the cluster of the posttest consisted of BACs 1 and 7, but at the retention test, BAC 3 was also connected with BACs 1 and 2, representing three of the four BACs of the preparation phase. A second cluster (same at the posttest) was related to the striking phase with BAC 6 (racket acceleration), BAC 8 (hitting point), and BAC 9 (wrist flap) of the final, swing phase. Lastly, a third functional cluster related to the final, swing phase included BAC 10 (forward bending of the body) and BAC 11 (racket follow-through).

With regard to the control group, the two cluster solutions of the pretest and the posttest were statistically different (λ = 0.53). When comparing to the expert structure, the mean dendrograms of the control group indicated minimal changes over time (pretest: ARI = 0.10; posttest: ARI = 0.15).

**FIGURE 10:**
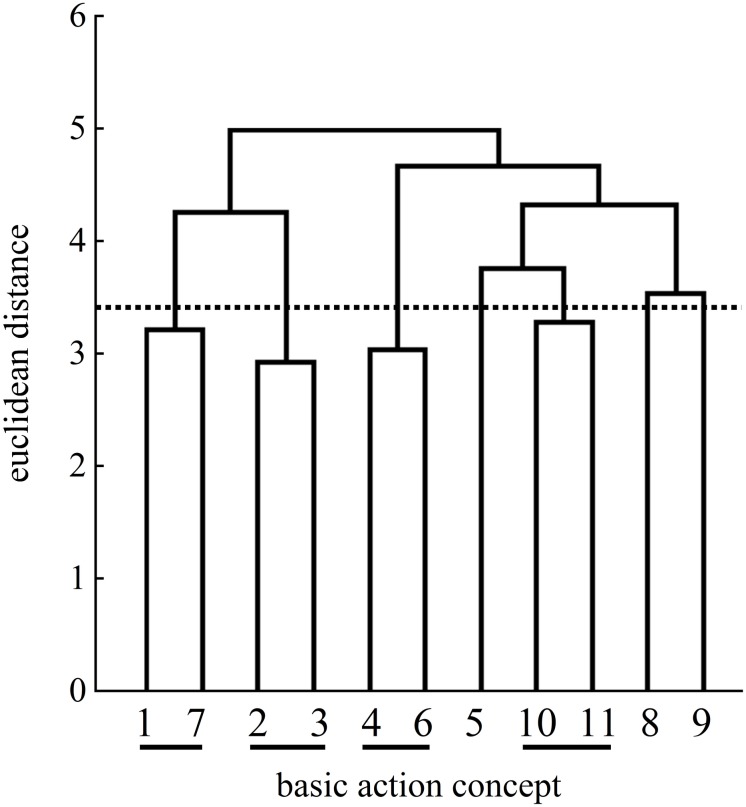
Mean group dendrogram of the control group’s (*n* = 14) tennis serves on the pretest.

**FIGURE 11:**
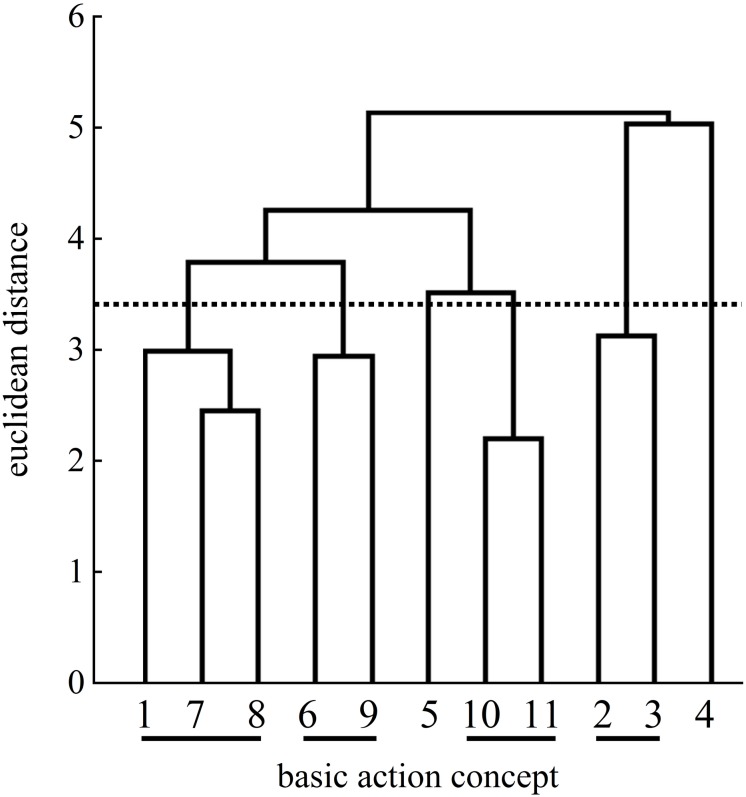
Mean group dendrogram of the control group’s (*n* = 14) tennis serves on the posttest.

At the posttest (Figure 11), the four clusters of the control group’s mean dendrogram showed two functional links, similar to pretest (Figure 10), related to the preparation phase with BAC 2 (forward movement of pelvis) and BAC 3 (bending the knees) as well as to the final, swing phase with BAC 10 (forward bending of the body) and BAC 11 (racket follow-through). A third cluster included BAC 1 (ball throw) of the preparation phase and BAC 7 (whole body stretch motion) and BAC 8 (hitting point) of the striking phase. The fourth cluster was related to the striking phase with BAC 6 (racket acceleration) and the final, swing phase with BAC 9 (wrist flap).

## Discussion

In the present field study, we examined the development of intermediate junior tennis players’ serve performance in terms of accuracy and velocity as well as the change in mental representation structures after a 5-week training period during which participants received analogy instructions, explicit instructions, or no instructions. Accounting for real-world conditions (Capio et al., 2019), we provided an equal amount of individual explicit and analogy instructions and implemented the study in the participants’ training sessions.

To sum up, findings demonstrated improvements regarding velocity from pretest to posttest for all participants and improved accuracy for participants in both instructed groups over time, but no significant group differences. Moreover, functional developments in mental representation structures were evident in both groups that received instructions, whereas this was not the case for the control group.

Findings of our study are in line with recent research that demonstrated improvements for analogy and explicit groups, but did not indicate significant performance differences between an analogy group and an explicit group on single tasks with adult novices (e.g., Schücker et al., 2010, 2013; Bobrownicki et al., 2019; Capio et al., 2019)^5^. Capio et al. (2019) examined novices and intermediate participants in a softball batting task. Players were assigned to four different groups, more precisely, an analogy intermediate group, an explicit intermediate group, an analogy novice group, and an explicit novice group. After six training sessions, batting performance was assessed. Capio et al. (2019) findings showed significant developments in the novice analogy group and the novice explicit group over time, while the authors found no significant development for the intermediate participants. Generally, results yielded no differences between the instructed groups (i.e., analogy and explicit) in novices as well as in intermediates. Our findings in terms of accuracy are in line with Capio et al. (2019) results for adult novices, while we found improvements for intermediate participants over time across both analogy and explicit group. Thus, it seems that both adult novices and junior athletes profit in a similar way from analogy and explicit instructions.

The results of the current study correspond as well as differ from those of a previous study examining intermediate junior table tennis players (Schlapkohl et al., 2012). Schlapkohl et al. (2012) showed benefits for explicit instructions in terms of hitting performance in a single task on the posttest, while results of the retention test, in line with the present results, indicated no significant differences between an analogy and an explicit group. Thus, their explicit group showed immediate benefits of (more) movement information compared to the analogy group that received only one analogy instruction.

However, it remains unclear whether the benefits of explicit instructions for the intermediate participants might be attributed to the amount or the type of the given instructions (Bobrownicki et al., 2018). Unlike (Schlapkohl et al., 2012) results, and providing the same amount of instructions, we showed that both types of instruction positively influence the outcome performance of intermediate tennis players. Assuming that intermediate junior tennis players are still in an associative stage of learning, the learner in this learning stage “starts to make more subtle adjustments in how the skill is performed” (Schmidt et al., 2018, p. 377). Although intermediate performance generally relies on more automatized processes and an increased degree of unconscious control, conscious processes are still important for the movement execution with the learners attending to specific parts of their movement. This might be the reason why both analogy and explicit instructions resulted in learning.

Indeed, and along these lines, Toner and Moran (2011) investigated the effects of conscious processing on golf putting proficiency (number of putts holed) and kinematics of expert athletes. More precisely, they compared the influence of conscious control (i.e., technical adjustments to a flawed aspect of their putting stroke) and conscious monitoring (i.e., club head impact spot) on expert golfers’ putting skills. Their results demonstrated that conscious control had no disruptive influence on expert golfers’ putting proficiency but did reduce the timing and the consistency of their strokes. In contrast, the conscious monitoring seemed to disrupt performance proficiency but had no influence on movement’s kinematics (Toner and Moran, 2011). These findings are in line with the present study demonstrating that conscious control of movements (with explicit or analogy instructions) had no disruptive influence on intermediate tennis players’ serve proficiency (i.e., accuracy and velocity) and highlight the importance of consciously attending to movement technique during associative stages of learning.

In the presented study, we included an additional control group that practiced the tennis serve without receiving the specific instructions. With respect to velocity and accuracy, we did not find group differences between the instructed groups and the control group, which might be related to the training conditions and the amount of practice. For the duration of the study, the same instructions have been used for each athlete. Moreover, we did not use any additional training equipment. This may have led to decreased motivation and may have prevented the potential benefits of instructions over uninstructed practice. Another interpretation concerns the amount of practice. The dose of practice under instructions might be not sufficient for further improvements in quantitative outcome performance as we investigated participants that already had experience in tennis serve.

Concerning mental representation structures of the tennis serve, the analogy group and the explicit group exhibited changes over the course of the study. The dendrograms of both the analogy group and the explicit group showed more meaningful clusters relating to the functional phases of the serve at the posttest than at the pretest. In addition, the analogy group and the explicit group showed the most functional links at the retention test compared to the other measurement points, indicating a development toward an expert mental representation structure.

Thus, practicing with analogy instruction or explicit instruction resulted in functional adaptations of mental representation structures. The results suggest that analogies and explicit instructions may help intermediate tennis players order mental representations of the tennis serve functionally, potentially leading to better performance over time in terms of accuracy.

The mental representation structure of the control group revealed minimal changes, but did not develop in a functional manner. Accordingly, these findings extend those of previous learning studies which showed that practice (Frank et al., 2013), practice with different foci (Land et al., 2014), or imagery training (Frank et al., 2014, 2018b) can structure mental representations. This study was the first to show that practicing with two common types of verbal instruction led to changes in mental representation structures.

In line with our results, Van Duijn et al. (2019a) also found no performance difference between an analogy and an explicit group examining novice adults in a hockey push-pass task, while in their study, results on a cognitive level showed positive developments for the analogy group. The authors suggested that an increased high-alpha power at the left temporal lobe may imply a reduction in verbal–cognitive processing for the analogy group. Thus, Van Duijn et al. (2019a) concluded that learning by analogies exhibited greater efficiency in verbal–cognitive processing compared to an explicit group possibly resulting from chunking processes.

In a recent study, Capio et al. (2019) also found no significant differences between two novice groups (explicit and analogy) as well as between two intermediate groups (explicit and analogy). As opposed to van Duijn, Hoskens, and Masters’ assumption, Capio et al. (2019) concluded that the intermediate players appear to have processed the analogies as new information and did not make links based on their existing knowledge. Unlike (Capio et al., 2019) conclusion and van Duijn, Hoskens, and Masters’ findings, in the present study with intermediate participants, we found functional developments in mental structures of both groups that received verbal instructions.

In our study, mental representation structure of the analogy group seemed to have become more similar to that of the expert mental representation structure at the retention test than that of the explicit group, as indicated by absolute ARI values. However, this cannot be interpreted as an advantage of the analogy group, since the two groups differed in their initial level of mental representation (and absolute ARI values). More specifically, the analogy group’s structure slightly developed from pretest to retention test (ΔARI = 0.04), while mental representation structure developed more from pretest to retention test in the explicit group (ΔARI = 0.38). Altogether, both groups’ mean dendrograms revealed more functional groupings (i.e., clusters of BACs that were part of the same functional phase of the movement) over the course of the study. Based on these results, we suppose that analogies as well as explicit instructions facilitate functional developments on a cognitive level as reflected by the structuring of representations through chunking processes. However, further research is needed in order to clarify differences in chunking processes between participants that received analogies or explicit instructions.

To conclude, this study is the first to show that mental representations of the tennis serve changed over the time during skill acquisition with explicit or analogy instructions. Although the intermediate tennis players in this study generally benefited from explicit and analogy instructions over time with regard to motor performance, the results did not demonstrate significant differences between the instructed groups. Thus, both types of verbal instructions led to changes in cognitive structures, whereas instructions’ cognitive benefits did not transfer one-to-one to improvements in performance (i.e., improvements over time across both groups in accuracy, but not in velocity). As such, the structuring of mental representation in motor memory may be a promising approach to uncover chunking processes during motor learning.

Future research may examine effects of verbal instructions in different sports’ fields measuring mental representation structures and outcome performances. To better understand the underlying processes and conditions of instruction-based learning, performance and mental representations should be investigated at individual levels considering an athlete’s individual characteristics.

## Data Availability Statement

The datasets generated for this study are available on request to the corresponding author.

## Ethics Statement

The studies involving human participants were reviewed and approved by Ethics Committee of Bielefeld University. Written informed consent to participate in this study was provided by the participants’ legal guardian/next of kin.

## Author Contributions

All authors conceived and designed the study, analyzed and interpreted the data, and drafted the manuscript. CM collected the data.

## Conflict of Interest

The authors declare that the research was conducted in the absence of any commercial or financial relationships that could be construed as a potential conflict of interest.

## Footnotes

1 The presented paper is based on the same sample described in Meier et al. (2019), but deals with different research questions and variables.

2 Velocity and accuracy are determining factors to assess tennis serve performance. Therefore, both components have been considered as an aggregate score for serve performance.

3 Serves in the net were not repeated. Thus, for the calculation of MRE and SRE, only those serves that landed behind the net were counted. The number of serves in the net was equal between and within the groups.

4 The results of the manipulation check have been adopted from Meier et al. (2019).

5 Bobrownicki et al. (2019) did not find improvements or differences between an analogy and an explicit group examining acute effects on motor control of a dart-throwing task in adult novices.
